# The Way of Distance Teaching Is Related to Adolescent Students’ Health and Loneliness during the School Closure in Finland

**DOI:** 10.3390/ijerph182312377

**Published:** 2021-11-25

**Authors:** Arja Rimpelä, Pirjo Lindfors, Jaana M. Kinnunen, Anna Myöhänen, Risto Hotulainen, Satu Koivuhovi, Mari-Pauliina Vainikainen

**Affiliations:** 1 Unit of Health Sciences, Faculty of Social Sciences, Tampere University, 33014 Tampere, Finland; pirjo.lindfors@tuni.fi (P.L.); jaana.kinnunen@tuni.fi (J.M.K.); anna.myohanen@tuni.fi (A.M.); 2 Department of Adolescent Psychiatry, Tampere University Hospital, P.O. Box 2000, 33521 Tampere, Finland; 3 Centre for Educational Assessment, Faculty of Educational Sciences, University of Helsinki, 00014 Helsinki, Finland; risto.hotulainen@helsinki.fi (R.H.); satu.koivuhovi@helsinki.fi (S.K.); mari-pauliina.vainikainen@tuni.fi (M.-P.V.); 4 Faculty of Education and Culture, Tampere University, 33014 Tampere, Finland

**Keywords:** distance learning, COVID-19, school closure, stress, health complaints, adolescent, transactional distance theory (TDT)

## Abstract

The COVID-19 pandemic enforced countries to close schools and rapidly transfer to distance teaching without preparation. Little is known about how different distance teaching practices influenced students’ wellbeing. We studied this during the period of school closures in Finland. Wellbeing was measured by health complaints and perceived loneliness, and distance learning was measured in terms of structure and dialogue of teaching, functioning of internet and digital equipment, difficulty of given tasks, and support for studies. All lower secondary schools were invited, and 29,898 students from 340 schools (grades 7–9) participated. A digital survey was distributed through schools just when these were reopened in May 2020. The main results were that the distance learning practices were related to adolescent health complaints and loneliness, so that less structure and dialogue in teaching, more problems with digital devices and internet, more difficult tasks and less support for studies were associated with higher health complaints and loneliness. From the point of view of students’ wellbeing, it matters how the distance learning is organised, how digital communication works, and if enough support for studies is available. These results of our research on distance learning practices during the present pandemic may guide schools in future crises and pandemic situations when distance learning is needed.

## 1. Introduction

In Spring 2020, the COVID-19 pandemic forced countries to close schools and implement distance learning practices without preparation. The disruptions in education supply are expected to result in significant setbacks for students, both in terms of learning loss and wellbeing. The almost overnight transition challenged otherwise a well-functioning Finnish education system and raised the concern of learning loss [1] and mental health problems [2] due to a variation in experience of distance teaching between schools and access to digital devices and online platforms. Pre-pandemic research on distance learning addresses the features and effects of planned distance learning activities, often in a higher education context but to a very limited extent even for the age group of the present study. However, the distance education described in the current study is crucially different from the previously reported approaches due to the sudden transition to it at a nationwide scale and the lack of experience and models for implementing it [1]. Little is known about how the ways of implementing distance learning affects students’ health.

COVID-19 prevention measures like school closure and social distancing were an unexpected stressor for a school community causing strain for its members and being a threat particularly for vulnerable children and parents [3,4]. Lack of school routines diminished outdoor activities, and poorly working online connections in teaching touched many students. The recent studies and narrative reviews have shown greater mental distress, anxiety, and risk of depression due to social isolation as well as increased family conflicts and violence between parents and children [3,5,6,7,8]. The effects on students’ health have also been seen in worsening health behaviours like decreasing physical activity or increasing screen time [7].

Prior studies have shown large differences in distance learning practices and learning support between Finnish schools during the school closure [9]. In this study, we examine, whether these differences were reflected in pupils’ wellbeing. Transactional Distance Theory (TDT) [10] has been used when analysing distance learning processes and practices. Structure and dialogue provide a basis for conceptualizing distance learning. In TDT, distance learning can be conceptualised by assessing the structure and dialogue of distance learning. According to TDT, a tighter structure and more regular dialogue between teachers and learners reduce a so-called transactional distance even in long physical distance. The longer the transactional distance, the more self-regulation is required from the learner. This means that older students are more capable of working in distance situations because of their more developed self-regulation competence [11].

In crisis-prompted distance learning situations, teachers’ feedback for learners is likely to be less personal than in ordinary teaching situations due to lack of experience in technology-mediated learning. When adding the missing face to face classmate support, some students are left out of guidance and monitoring leading to a decreasing school engagement. During the school closure, distance learning took place mainly in students’ home. Many Finnish parents were at home, but they were working remotely, giving them a limited possibility to support their children during the school day even if they had the competence for it [12].

The transition to unexpected distance learning seems to be easy for some students, while for others it is not that straightforward because the adjustment to digital environment requires more self-regulated learning (see [13]). Self-regulated learning is not only a feature of a student but also depends on teachers’ way of teaching. Cai et al. [14] showed that when self-regulated learning is required, students do worse in a more passive learning such as watching teachers live on the internet than when using protocol-guided learning to support self-regulated learning. Large differences in distance learning practices and learning support was observed between Finnish schools during the school closure [9].

Our aim is to study the influence of implementation of distance learning practices in terms of structure and dialogue on 13–16-year-old adolescent students’ wellbeing, namely health complaints and perceived loneliness during the two-month school closure in March–May 2020. The context of our study is Finland which is well-known of its success in international large-scale PISA studies (The Programme for international Student Assessment) since the publication of the first results in 2001, and of its equal educational system with small between-school variation [15,16]. The schools were reopened for two weeks in May before the end of the spring term, during which time our survey was conducted.

## 2. Materials and Methods

### 2.1. Participants

Local and national education authorities were contacted in April 2020. A collaboration agreement was made first with 16 municipalities in Southern Finland, after which the Ministry of Education and Culture supported the extension to the whole country. Research permits were obtained for the 16 original municipalities and from the Ministry for the rest of the country. School leaders (i.e., principals) were contacted for school-level research permits. Schools from large cities were more reluctant to participate due to stricter municipal-level policies concerning research participation.

The data were collected online using digital survey system (Qualtrics) during the last two weeks of the school year in May when the schools were reopened. The links to the electronic questionnaire were delivered to students through school principals using the usual communication channels of the school. The participation was voluntary, and the parents could prevent their child from participating. Most pupils answered the questionnaire during school hours, but it was possible to answer the questionnaire at home. The data were collected anonymously, but respondents were informed that school and municipality identification codes were included in the response.

The Finnish basic education system consists of primary grades 1–6 (age 7–13), lower secondary grades 7–9 (age 13–16) and the optional grade 10, which very few students attend before transferring to upper secondary education. The pupil questionnaire was distributed to grades 4 to 10. Altogether 61,974 pupils responded (approximately 10% of the pupil population). The pupils were from 886 different schools representing 41% of the 2187 basic education schools. Thus, the non-response occurred largely at school level due to principals not distributing the survey. The participating schools were in 226 out of 310 municipalities (73%), which means that the sample can be interpreted as nationally representative. Questions on health complaints were presented only to students of 7th–10th grades. In this study we included only those participants from the grades 7 to 9 of Finnish-speaking schools (N = 31,261), who had studied at home during the school closure (N = 29,898). Of those, 14 respondents were excluded due to missing gender information, leaving N = 29,884. The final sample included answers from 159 municipalities and 340 schools. The sample distributions are presented by gender in Table 1.

### 2.2. Health Variables

Three outcome variables were formed: daily health complaints, weekly health complaints, and loneliness. Pupils were asked if they had the following complaints during the lockdown: (1) neck, shoulder, or back pain, (2) headache, (3) difficulties in falling asleep or getting awake night-time, (4) tiredness or exhaustion, (5) low mood or depression, or (6) difficulties with concentration. These symptoms have been widely used in youth studies to measure wellbeing, e.g., Health Behaviour in School-Aged Children where validity has been shown adequate and the test-retest reliability good [17]. The symptoms are perceived, and they are not supposed to be diagnostic criteria. The underlying assumption is that at this age children can assess and report their symptoms and feelings reliably. For each symptom the options were: seldom or not at all, approximately once a month, approximately weekly, daily. If a participant had given an answer to at least one of the questions, missing answers in the other symptoms were replaced with value “seldom or not at all”. If none of the symptoms was answered, the respondent was excluded from the analyses (N = 4087) giving the final sample size N = 25,797. Cronbach’s alpha of the six health complaint questions was 0.84 which is on a very good level. For the analyses, two sum variables were composed. The variable daily health complaints was the sum of the daily symptoms in the six questions (range 0–6). Due to a very skewed distribution, the variable was classified into two categories: no daily symptoms; one or more symptoms. The variable weekly health complaints was correspondingly the number of the symptoms appearing weekly or more often (range 0–6). The variable was classified into three categories: no symptoms, one or two symptoms, three or more symptoms. The participants were asked if they had felt lonely during the lockdown with three options: never, sometimes, often. This question was answered by 26,141 respondents. The number of respondents who had answered stress symptoms and loneliness was 25,730.

Gender was queried in the beginning of the survey. Three options were given (girl, boy, other).

### 2.3. Distance Learning Variables

According to the TDT theory, the distance learning variables were divided into two categories, those measuring the structure of teaching and those measuring the dialogue between teacher and student. In addition, a question on the functioning of the internet connection was included as well as support for schoolwork compared to normal times.

Structure was measured by two questions, namely following the schedule and perceived difficulty of the tasks. The question on how the schedule was followed during the exceptional period had three options: all the time, partially, not at all. The question on difficulty of the tasks compared to normal times had five options: clearly easier, somewhat easier, as easy or as difficult, somewhat more difficult, clearly more difficult. For the analyses, the variable was reclassified into three 1 = clearly or somewhat easier, 2 = same as earlier, 3 = clearly or somewhat more difficult.

Dialogue was measured with two questions, whether the teacher taught through video and if the teacher was available through video, chat or in some other way during the classes or other scheduled times. The question on teaching through video was repeated for mother tongue, mathematics and the first foreign language with the options: 1 = every lesson, 2 = most lessons, 3 = now and then, 4 = seldom, 5 = not at all. We computed a mean of these for each respondent. If the answer for one item was missing, two others were used. The respondent was excluded if there was only one answer or none. To make the results easier to read, the means were classified into four categories: all or most lessons (means from 1 to 1.50), means from 1.51 to 2.50, means from 2.51 to 3.67, rarely or not at all (means from 3.68 to 5.00). Teacher availability was asked correspondingly for the three school subjects with the options: 1 = always according to the time schedule, 2 = most of the time, 3 = now and then, 4 = seldom, 5 = not at all. Means of the answers were computed similarly as teaching through video. For the analyses, the means were classified into four categories: always according to the time schedule (means 1.00 to 1.32), means from 1.33 to 1.99, means from 2.00 to 2.99, occasionally or seldom according to the time schedule (means from 3.00 to 5.00).

Functioning of the digital devices and internet connections was measured with two questions on possible difficulties with internet connection or IT devices and sharing digital devices with family members. The question on difficulties with devices or internet connections had five options: daily, several times a week, approximately once a week, once a month, not at all. In the analyses the scale was turned. Sharing devices with family members was measured by a question of need to take turns with your siblings or parents when using devices, with the options yes and no. Perceived support for studies compared to normal situation had four options: clearly less, somewhat less, as much as earlier, somewhat more, clearly more. For the analyses, the variable was coded into three classes, more support, as much as earlier, or less support.

The scale for distance learning was developed for the present study based on transactional distance theory and it is described in detail in Section 2.3. No existing scale addressed the implementation of distance learning in basic education in the context of an unexpected crisis and there is very little research overall regarding distance learning of children and youth. Therefore, a new scale had to be developed based on a well-established theory that has been previously used mainly in adult education and higher education context.

### 2.4. Statistical Methods

In educational contexts, pupils are nested within schools and it is a standard procedure to check the group level effects. Very often the school level needs to be taken into account when conducting the actual analyses so that the results are not biased. Given that school level effects were possible, these were investigated by computing intraclass correlation (ICC) and design effect for each of the three outcome variables, using school as a grouping variable. For the weekly health complaints ICC = 0.008; N = 25,797; design effect = 1.7, for the daily health complaints ICC = 0.007; N = 25,797; design effect = 1.6, for loneliness ICC = 0.008; N = 26,141; design effect = 1.7. The ICC values were so low that adjusting for school was omitted in the analyses [18].

We used logistic regression modelling for analysing the effect of each explanatory variable on the number of daily stress symptoms. Due to the three-class nature of the outcomes, we conducted corresponding analyses for weekly stress symptoms and loneliness by multinomial logistic regression. Odds ratios (OR) were computed for (1) each explanatory variable as the only predictor, adjusting for gender and (2) all other significant predictors, including gender, adjusting each of the reported explanatory variables. Statistically significant results were *p* < 0.05.

All analyses were performed using IBM SPSS software version 25 (IBM Corp., Armonk, NY, USA).

## 3. Results

Table 1 presents the distributions of health variables and Table 2 the distributions of distance learning variables.

### 3.1. Health Complaints

The results of the multinomial logistic regression analyses are presented in Table 3. The group with no weekly symptoms were used as the reference group (OR = 1.0). In the binary analyses adjusted for gender, Model 1, ORs for weekly health complaints were significantly higher for all measured variables except for sharing digital equipment with family members when compared to the reference group. The ORs increased with decreasing structure, dialogue, and support for studies and with increasing problems with internet or digital devices. The ORs for the group with 3–6 weekly complaints were higher than for the group with 1–2 complaints. The highest ORs were observed for the group with 3–6 complaints when tasks were more difficult compared to normal (OR = 3.0) and when the child had daily problems with internet or digital equipment (OR = 5.8). In adjusted models with all significant variables, the ORs became smaller but only teaching through video lost its significance.

The results of having at least one daily health complaint are presented in Table 4. The results in both binary (Model 1) and adjusted (Model 2) analyses mainly followed the results of weekly health complaints. The exception was that teaching through video was not related to health complaints in the binary analysis but in the adjusted analysis less teaching with video was related to less symptoms. Unlike weekly health complaints, sharing digital equipment with family was associated with less daily health complaints.

### 3.2. Loneliness

The results of perceived loneliness are presented in Table 5. The group that did not report loneliness was used as the reference group. In the binary analyses, all measured variables were related to loneliness and in general, the ORs were higher for those that reported loneliness often than those who reported sometimes. The ORs increased with decreasing structure, dialogue, and support for studies and with increasing problems with internet or digital devices and unlike in health complaints, also with sharing digital equipment with family. In the adjusted analyses, the ORs became smaller, and the significance disappeared for the variable ‘teaching through video’ and partly for ‘teaching schedule was followed’—the latter only for those who reported loneliness often.

## 4. Discussion

We studied implementation of distance learning on 13–16-year-old adolescent students’ wellbeing in terms of health complaints and perceived loneliness. The main result was that the way distance learning was implemented was associated with both wellbeing indicators. Less structure and dialogue in teaching, less support for studies, problems with digital devices, and difficult tasks were associated with increased health complaints and perceived loneliness.

Knowledge on the association between implementation of distance learning and adolescent students’ wellbeing is scarce. Before the COVID-19 pandemic, distance learning was hardly used in schools with adolescent students and if it was, not in such a large scale like in 2020.

Even though there are no directly comparable studies to our findings, earlier studies on academic stress and the psychosocial work environment in school seem to support our findings. School-related stress measured by difficulties in schoolwork and heavy load of schoolwork has been shown to increase students’ health complaints while teachers support has an opposite influence [19]. The relationship between health complaints and school demands is not only an individual phenomenon but also high demands on class level predict higher health complaints among students [20]. In our study, students who reported that tasks were more difficult than normally reported more symptoms and those who perceived less support for studies had more symptoms. Unexpectedly, those getting more support than normal also reported more symptoms. The explanation may be that these students had difficulties that stressed them, but they had people around who supported them in this exceptional situation more than normal.

The role of psychosocial working environment on adolescents’ somatic health complaints was shown by Sonmark and Modin [21]. Using the demand-control-support model [22], they studied decision control in school class and social support from teachers, parents, and peers as a stress-moderating resource. The decision control meant students’ role in deciding activities in class and how class time was used. Both higher perceived decision control and higher support were related to better health. We studied health complaints and loneliness during the crisis-prompted distance teaching during the Spring 2020 school closure using transactional distance theory [9] and found problems in structure of teaching, in dialogue between teachers and students as well as in learning support and internet connections. These were related to increased health complaints and loneliness.

The results can be at least partly explained by demand-control-support model [22]. Adapted to school context, it would mean the increased demands put on students in learning situations as well as their feeling of less control over those as well as diminished support for learning. Distance teaching and adjustment to digital environment increased the demands for self-regulated learning. Self-regulated learning skills vary individually and are related to age and developmental stage of the child but also how distance teaching is implemented (e.g., [13]). Although some students do well in the situation, for the others the demands feel overwhelming, particularly when teachers are not available on scheduled lesson times, timetable are not followed, internet connections perform inadequately, and support for studies is less than normal. The above results suggest that for some students, control over own schoolwork diminished at least during the lessons, while it could have increased among students who had good self-regulating learning skills because they could e.g., plan how to conduct their learning tasks, easily navigate between learning applications and select time of doing homework. It has to be noted that in spite of the challenging situation and teachers’ and schools’ lack of experience in distance teaching, the Finnish national education authorities did not have an official permission to lower the target learning level expressed in the national curriculum.

Video teaching was among the core practices during the school closure. Even though it was related to the wellbeing indicators in binary models, the significance disappeared when other distance learning variables were adjusted for. This can be interpreted so that it is not so important if teaching is mainly through video or not, but that teaching is structured and that dialogue between students and teachers is available and even more important that internet connections and digital devices work properly.

The digital devices and internet connections were often inadequate in schools and in some students’ and teachers’ home and all teachers did not have necessary skills to use programmes or devices, particularly in the beginning of the exceptional period [14]. That is why it is understandable that these problems were stressors during the distance learning causing increase in students’ health complaints.

Strengths and limitations. Our dataset was large consisting of participants from schools all over the country. Even though all schools and every student in the school did not participate, the results can be generalized nationally. Only pupils on lower secondary grades were studied even though the pandemic has most likely influenced the wellbeing of primary school pupils as well. However, in the primary grades we had to use a shortened version of the questionnaire which did not include health complaints. All Finnish schools have easy access to digital systems (e.g., via mobile phones) for information exchange between school personnel, parents, and students. Nevertheless, there may have been some students, for whom the quality of mobile phones and computers limited answering to the digital survey. Our survey was conducted during the last two weeks of the Spring term when schools were reopened. This was also the time of the survey meaning that there is a small retrospective element. In that case, this will most like diminish the observed effects.

## 5. Conclusions

Future pandemics and other crises may generate need for school closures and transfer to distance learning in schools. From the point of view of students’ health and loneliness, it matters how distance learning is implemented. Well-structured teaching (e.g., teaching schedule is followed), dialogue between students and teachers (e.g., teacher available on scheduled times), social support for studies, and well-functioning internet connections and devices promote students’ health and decrease loneliness during distance learning periods.

## Figures and Tables

**Table 1:** Distributions of the health variables by gender.

Health	Boys		Girls		Other		Total	
	N	%	N	%	N	%	N	%
Weekly health complaints								
No	4513	43.6	3190	21.3	89	18.7	7792	30.2
1–2	3366	32.5	4717	31.5	107	22.4	8190	31.7
3–6	2468	23.9	7066	47.2	281	58.9	9815	38.0
Total	10,347	100.0	14,973	100.0	477	100.0	25,797	100.0
Daily health complaints								
No	8043	77.7	8973	59.9	207	43.4	17,223	66.8
At least one	2304	22.3	6000	40.1	270	56.6	8574	33.2
Total	10,347	100.0	14,973	100.0	477	100.0	25,797	100.0
Loneliness								
No	6148	58.6	4789	31.6	172	35.3	11,109	42.5
Sometimes	3510	33.5	7537	49.7	192	39.4	11,239	43.0
Often	829	7.9	2841	18.7	123	25.3	3793	14.5
Total	10,487	100.0	15,167	100.0	487	100.0	26,141	100.0

**Table 2:** Distributions of the explanatory variables.

Distance Learning during the School Closure	N	%
Structure		
Teaching schedule was followed		
All the time	11,553	41.8
Partly	14,180	51.3
Not at all	1882	6.8
Total	27,615	100.0
Tasks compared to normal		
Easier	3463	12.5
Same as earlier	13,364	48.4
More difficult	10,769	39.0
Total	27,596	100.0
Dialogue		
Teaching through video		
All or most lessons (1.00–1.50)	3399	12.8
1.51–2.50	9194	34.7
2.51–3.67	9938	37.5
Rarely or not at all (3.68–5.00)	3938	14.9
Total	26,469	100.0
Teacher available on schedule times		
Always (1.00–1.32)	7901	29.9
1.33–1.99	6956	26.4
2.00–2.99	8200	31.1
Occasionally or seldom (3.00–5.00)	3341	12.7
Total	26,398	100.0
Digital devices and support		
Problems with internet or equipment		
Not at all	10,037	34.5
Monthly	6830	23.5
Weekly	10,479	36.0
Daily	1728	5.9
Total	29,074	100.0
Support for studies compared to normal		
More	3100	11.3
Same as earlier	15,251	55.4
Less	9175	33.3
Total	27,526	100.0

**Table 3:** Effects of distance learning variables on weekly health complaints. Results of multinomial logistic regression modelling. Odds ratios (ORs) of bivariate and multivariate models. Significant variables in bold.

Distance Learning during the School Closure	Number of Weekly Health Complaints			
	1–2	3–6	1–2	3–6
	OR ^1^	OR ^1^	OR ^2^	OR ^2^
Structure				
Teaching schedule was followed				
All the time	1.0	1.0	1.0	1.0
Partly	**1.2**	**1.4**	**1.1**	**1.3**
Not at all	**1.3**	**2.2**	**1.2**	**1.8**
Tasks compared to normal				
Easier	1.0	1.0	1.0	1.0
Same	1.0	**0.9**	1.1	0.9
More difficult	**1.8**	**3.0**	**1.7**	**2.5**
Dialogue				
Teaching through video				
All or most lessons (1–1.50)	1.0	1.0	1.0	1.0
1.51–2.50	1.0	**1.1**	0.9	0.9
2.51–3.67	1.1	**1.2**	1.0	1.0
Rarely or not at all (3.68–5.00)	1.1	**1.2**	1.0	**0.9**
Teacher available on schedule times				
Always (1.00–1.32)	1.0	1.0	1.0	1.0
1.33–1.99	**1.2**	**1.3**	**1.2**	**1.2**
2.00–2.99	**1.3**	**1.6**	**1.2**	**1.2**
Occasionally or seldom (3.00–5.00)	**1.4**	**2.1**	**1.2**	**1.5**
Digital devices and support				
Problems with internet or equipment				
Not at all	1.0	1.0	1.0	1.0
Monthly	**1.2**	**1.1**	**1.2**	**1.1**
Weekly	**1.6**	**2.4**	**1.5**	**2.1**
Daily	**2.2**	**5.4**	**1.9**	**3.8**
Share digital equipment with family				
No	1.0	1.0	-	-
Yes	1.0	1.0	-	-
Support for studies compared to normal				
More	1.0	1.0	1.0	1.0
Same	**0.7**	**0.6**	**0.8**	**0.7**
Less	**1.3**	**1.8**	**1.2**	**1.6**

^1^ Model 1. Each variable at a time, adjusted for gender, ^2^ Model 2. All significant variables in one model, adjusted for gender.

**Table 4:** Effects of distance learning variables on daily health complaints. Results of logistic regression modelling. Odds ratios (OR) of bivariate and multivariate models. Significant variables in bold.

Distance Learning during the School Closure	At Least One Daily Health Complaint	
	OR ^1^	OR ^2^
Structure		
Teaching schedule was followed		
All the time	1.0	1.0
Partly	**1.2**	**1.1**
Not at all	**2.1**	**1.8**
Tasks compared to normal		
Easier	1.0	1.0
Same	**0.8**	**0.8**
More difficult	**2.0**	**1.8**
Dialogue		
Teaching through video		
All or most lessons (1–1.50)	1.0	1.0
1.51–2.50	1.0	**0.9**
2.51–3.67	1.1	**0.9**
Rarely or not at all (3.68–5.00)	1.1	**0.8**
Teacher available on schedule times		
Always (1.00–1.32)	1.0	1.0
1.33–1.99	**1.2**	**1.1**
2.00–2.99	**1.4**	**1.1**
Occasionally or seldom (3.00–5.00)	**1.8**	**1.4**
Digital devices and support		
Problems with internet or equipment		
Not at all	1.0	1.0
Monthly	**0.9**	**0.9**
Weekly	**1.5**	**1.3**
Daily	**3.4**	**2.6**
Share digital equipment with family		
No	1.0	1.0
Yes	**0.9**	**0.8**
Support for studies compared to normal		
More	1.0	1.0
Same	**0.7**	**0.7**
Less	**1.5**	**1.4**

^1^ Model 1. Each variable at a time, adjusted for gender, ^2^ Model 2. All significant variables in one model, adjusted for gender.

**Table 5:** Effects of distance learning variables on perceived loneliness. Results of multinomial logistic regression modelling. Odds ratios (OR) of bivariate and multivariate models. Significant variables in bold.

Distance Learning during the School Closure	Perceived Loneliness			
	Sometimes	Often	Sometimes	Often
	OR ^1^	OR ^1^	OR ^2^	OR ^2^
Structure				
Teaching schedule was followed				
All the time	1.0	1.0	1.0	1.0
Partly	**1.2**	**1.3**	**1.1**	1.1
Not at all	1.0	**1.6**	**0.9**	1.1
Tasks compared to normal				
Easier	1.0	1.0	1.0	1.0
Same	1.0	**0.9**	1.1	1.0
More difficult	**1.5**	**2.5**	**1.4**	**2.1**
Dialogue				
Teaching through video				
All or most lessons (1–1.50)	1.0	1.0	1.0	1.0
1.51–2.50	**1.1**	1.1	1.0	0.9
2.51–3.67	**1.2**	**1.2**	1.0	1.0
Rarely or not at all (3.68–5.00)	**1.2**	**1.4**	1.1	1.1
Teacher available on schedule times				
Always (1.00–1.32)	1.0	1.0	1.0	1.0
1.33–1.99	**1.2**	**1.5**	**1.1**	**1.3**
2.00–2.99	**1.3**	**1.6**	**1.2**	**1.3**
Occasionally or seldom (3.00–5.00)	**1.4**	**2.4**	**1.2**	**1.6**
Digital devices and support				
Problems with internet or equipment				
Not at all	1.0	1.0	1.0	1.0
Monthly	**1.4**	**1.1**	**1.4**	**1.1**
Weekly	**1.8**	**2.0**	**1.6**	**1.7**
Daily	**1.7**	**4.2**	**1.4**	**2.8**
Share digital equipment with family				
No	1.0	1.0	1.0	1.0
Yes	**1.2**	**1.3**	**1.1**	**1.2**
Support for studies compared to normal				
More	1.0	1.0	1.0	1.0
Same	**0.8**	**0.6**	**0.9**	**0.7**
Less	**1.5**	**2.1**	**1.4**	**1.9**

^1^ Model 1. Each variable at a time, adjusted for gender, ^2^ Model 2. All significant variables in one model, adjusted for gender.

## Data Availability

The data presented in this study are available on request from the authors with a research plan and a signed contract with Tampere University and the University of Helsinki.
